# Integrative network analysis identifies candidate genes shared between ferroptosis and cuproptosis pathways in rheumatoid arthritis pathogenesis

**DOI:** 10.1371/journal.pone.0358176

**Published:** 2026-09-11

**Authors:** Abbas Ahmad, Shehzad Khalil, Douglas Law, Patricio R. De los Ríos-Escalante, Mostafa A. Abdel-Maksoud, Saeedah Almutairi, Aljawharah Fahad Alabbad, Waheed Ahmad, Ayaz Ahmad

**Affiliations:** 1 Department of Biotechnology, Abdul Wali Khan University Mardan, Khyber Pakhtunkhwa, Pakistan; 2 Institute of Biotechnology and Genetic Engineering, The University of Agriculture Peshawar, Peshawar, Khyber Pakhtunkhwa, Pakistan; 3 Faculty of Health and Life Sciences, INTI International University, Nilai, Negeri Sembilan, Malaysia; 4 Departamento de Ciencias Biológicas y Químicas, Facultad de Recursos Naturales, Universidad Católica de Temuco, Temuco, Chile; 5 Núcleo de Estudios Ambientales, Facultad de Recursos Naturales, Universidad Católica de Temuco, Temuco, Chile; 6 Research Chair of Biomedical Applications of Nanomaterials, Biochemistry Department, College of Science, King Saud University, Riyadh, Saudi Arabia; 7 Botany and Microbiology Department, College of Science, King Saud University, Riyadh, Saudi Arabia; 8 Minnesota Dental Research Center for Biomaterials and Biomechanics, School of Dentistry, University of Minnesota, Minneapolis, Minnesota, United States of America; UFRN: Universidade Federal do Rio Grande do Norte, BRAZIL

## Abstract

Rheumatoid arthritis (RA) is a chronic autoimmune inflammatory disease characterized by synovial inflammation, progressive joint destruction, and systemic immune dysregulation. Recent findings suggest that disturbed metal homeostasis and regulated cell death pathways, including ferroptosis (iron-dependent lipid peroxidation) and cuproptosis (copper-dependent mitochondrial proteotoxic stress), contribute to RA pathogenesis. In this study, we used an integrative bioinformatic approach combining weighted gene co-expression network analysis (WGCNA), differential expression analysis, functional enrichment, protein-protein interaction (PPI) network construction, and immune cell infiltration deconvolution to identify key metal-dependent cell death regulators in RA. Using bulk RNA-seq data from peripheral CD14^+^ monocytes (GSE294225) from 15 healthy controls and 9 patients with active RA (DAS28 > 2.7), we identified 1,410 significantly differentially expressed genes (DEGs) (adjusted P < 0.05, log2 fold change > 1). WGCNA revealed an RA-associated module enriched in oxidative stress, mitochondrial dysfunction, and cell death pathways. Overlap analysis of ferroptosis- and cuproptosis-related gene sets distinguished three upregulated hub genes, including *FTH1* (*ferritin heavy chain 1*), *SOD2* (*superoxide dismutase 2*), and *CDKN2A* (*cyclin-dependent kinase inhibitor 2A*) as key candidate regulators. These genes showed high module membership, significant differential expression in RA monocytes, and notable associations with immune infiltration patterns, including increased pro-inflammatory monocytes/macrophages and reduced regulatory T cells. Functional enrichment also highlighted oxidative stress response, iron and copper homeostasis, mitochondrial respiration, and cellular senescence. The single-cell analysis further showed that these hub genes are predominantly expressed in the RA synovial macrophages and fibroblasts, two major mediators of joint pathology. Together, these findings indicate that there may be an association between ferroptosis-related pathways and cuproptosis-related pathways in RA and suggest that *FTH1*, *SOD2*, and *CDKN2A* are candidate biomarkers and candidate therapeutic targets.

## 1. Introduction

Rheumatoid arthritis (RA) is a chronic, systemic inflammatory autoimmune disorder that primarily affects synovial joints, leading to progressive cartilage and bone destruction, functional disability, and an increased risk of mortality [1]. RA also imposes a substantial socioeconomic burden. In 2019, its global prevalence was estimated at 18 million cases, and the global age-standardized incidence rate was approximately 13.48 per 100,000 population in 2021 [2]. The disease shows a clear female predominance, with nearly 70% of the cases occurring in women, and it is more commonly reported in industrialized countries, possibly due to demographic factors, environmental exposures, and underdiagnosis in low- and middle-income regions [2]. The burden of RNA is projected to rise further by 2040, particularly among young adults (20–54 years) in lower-income settings, underscoring the need for a deeper understanding of its underlying mechanisms [3].

The pathogenesis of RA is highly complex and involves genetic susceptibility, environmental triggers such as smoking and infections, and dysregulation of both innate and adaptive immune responses, ultimately resulting in persistent synovial inflammation and extra-articular manifestations, including cardiovascular and pulmonary complications [4]. A central role is played by fibroblast-like synoviocytes (FLS), which acquire an aggressive, tumor-like phenotype, promote pannus formation, and secrete pro-inflammatory cytokines such as *TNF-α* and IL-6, thereby contributing to matrix degradation and joint damage [5]. In addition, T cells, B cells, macrophages, and neutrophils infiltrate the synovium and sustain a pro-inflammatory microenvironment by producing autoantibodies, including rheumatoid factor and anti-citrullinated protein antibodies, and by activating osteoclasts that drive bone erosion [6]. More recent studies have further highlighted the contributions of epigenetic alterations and metabolic reprogramming and have clarified how environmental influences interact with inherited risk factors, such as *HLA-DRB1* alleles, to initiate and perpetuate disease progression [7].

Recent advances in multi-omics technologies and bioinformatics have shown that non-apoptotic regulated cell death (RCD) pathways may also play important roles in RA pathogenesis [8]. Apoptosis, a caspase-dependent process, is a key mechanism for maintaining immune homeostasis; however, resistance to apoptosis in FLS contributes to synovial hyperplasia [9]. Increasing evidence points to alternative RCD pathways, particularly ferroptosis and cuproptosis. Ferroptosis is an iron-dependent form of cell death characterized by lipid peroxidation, glutathione depletion, and inactivation of glutathione peroxidase 4 (GPX4), whereas cuproptosis is a copper-mediated form of cell death associated with mitochondrial proteotoxic stress, aggregation of lipoylated proteins, and loss of iron-sulfur cluster protein [10,11]. These pathways are especially relevant to RA, a disease strongly linked to oxidative stress, in which elevated reactive oxygen species (ROS), chronic synovial inflammation, and disturbed metal ion homeostasis, including iron overload and copper imbalance, may collectively promote cell injury, immune activation, and joint destruction [12]. Ferroptosis has been implicated in regulating FLS proliferation, chondrocyte death, and inflammatory responses in RA, and inflamed joints have been reported to exhibit altered iron distribution and increased lipid peroxidation [13]. Likewise, emerging evidence links copper-dependent toxicity and cuproptosis to RA progression, suggesting that mitochondrial dysfunction and proteotoxic stress in immune cells and synoviocytes may contribute to disease development, particularly in the context of the abnormal copper levels observed in patients with RA [11]. Taken together, the potential overlap between ferroptosis and cuproptosis through shared features such as mitochondrial dysfunction and disrupted metal homeostasis represents a promising and still underexplored framework for understanding the oxidative and metabolic basis of RA [14].

Integrative bioinformatic approaches are well-suited to dissect such complex biological interactions by combining transcriptomic and other high-dimensional datasets. Weighted gene co-expression network analysis (WGCNA) is a powerful systems biology method that constructs scale-free gene co-expression networks, enabling the identification of gene modules associated with clinical traits and of hub genes with potential biomarker or therapeutic relevance in RA [15]. Differential expression analysis using tools such as DESeq2 further helps identify genes with significant transcriptional alteration between RA and healthy samples [16]. In parallel, immune profiling using single-sample gene set enrichment analysis (ssGSEA) can estimate immune cell infiltration and functional status of immune cell populations from bulk RNA data, providing insight into immune signatures associated with disease activity in RA synovium or peripheral blood [17]. These strategies have already been successfully applied in RA research to characterize immune infiltration patterns, including increased macrophages and neutrophils, and to link these patterns to pathologic pathways [18]. However, the combined contribution of ferroptosis- and cuproptosis-related genes to RA, particularly in relation to immune dysregulation, remains insufficiently understood.

In the present study, we used an integrative bioinformatics framework to investigate the role of metal-dependent regulated cell death in RA. Using the GSE294225 dataset, we first identified differentially expressed genes (DEGs), then applied WGCNA to detect disease-associated modules and hub genes. Ferroptosis- and cupeorptosis-related regulators were subsequently screened, and immune cell infiltration was assessed using ssGSEA. Finally, we explored the relationships between candidate genes and immune signatures to identify candidate biomarkers and candidate therapeutic targets associated with RA pathogenesis.

## 2. Materials and methods

**Fig 1:**
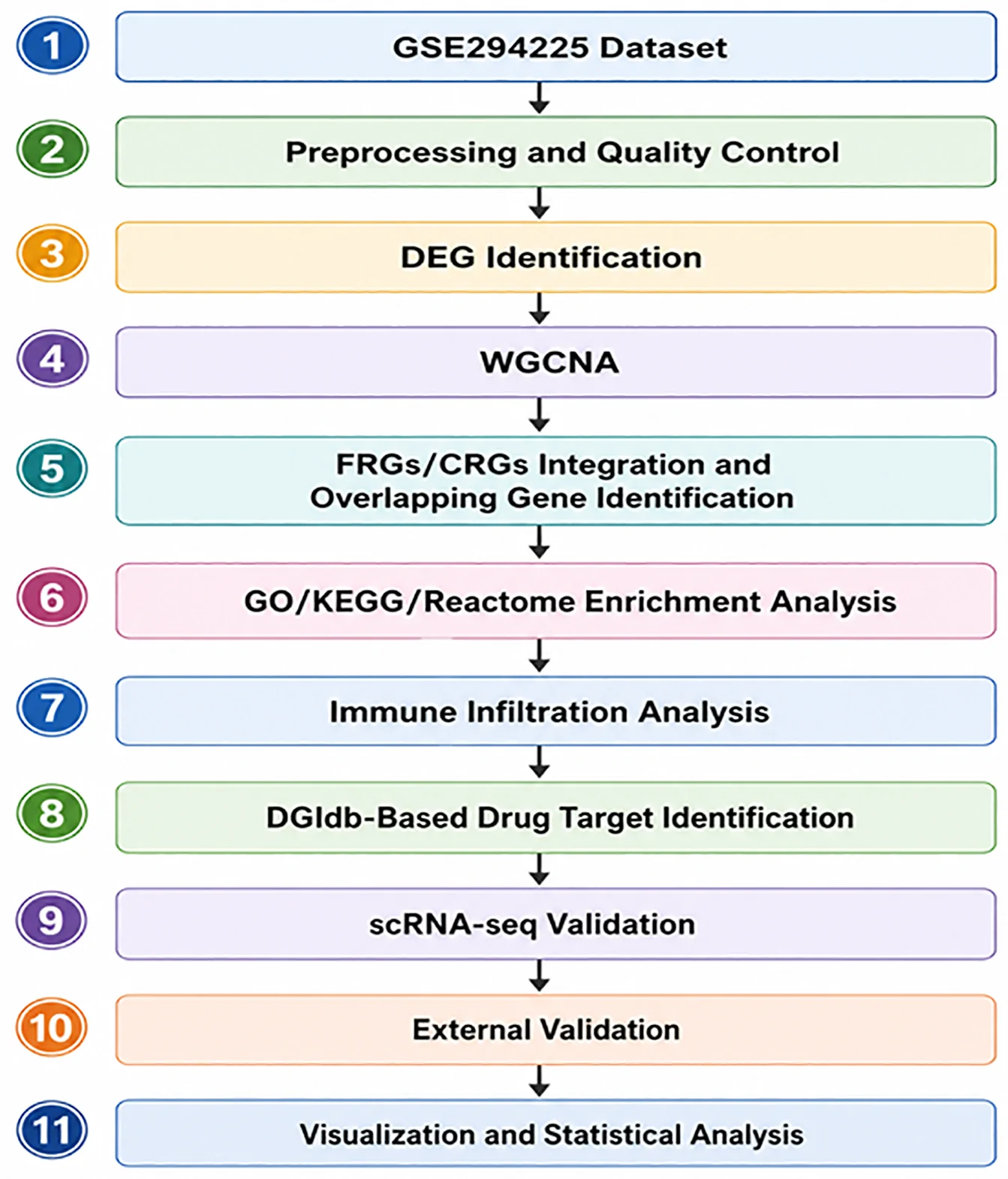
Flowchart of the current study.

Fig 1 shows stepwise overview of the bioinformatics workflow employed in this study: (1) retrieval of the GSE294225 rheumatoid arthritis dataset and preprocessing, including quality control and normalization; (2) identification of differentially expressed genes (DEGs); (3) weighted gene co-expression network analysis (WGCNA) to identify key gene modules; (4) integration of ferroptosis-related genes (FRGs) and cuproptosis-related genes (CRGs) to identify overlapping genes; (5) functional enrichment analyses, including GO, KEGG, and Reactome pathways; (6) immune infiltration analysis; (7) identification of potential therapeutic targets using DGIdb; (8) single-cell RNA sequencing (scRNA-seq) analysis; (9) external validation using independent datasets; and (10) visualization and statistical analysis of the results.

### 2.1. Dataset and preprocessing

The Bulk RNA-sequencing data were obtained from the Gene Expression Omnibus (GEO) under the accession GSE294225, which contained 24 samples: 15 healthy controls (HC) and 9 patients with active rheumatoid arthritis (RA; DAS28 > 2.7). These samples are representative of CD14^+^ peripheral blood monocytes of the whole blood [19,20]. The initial count- matrix of 18,205 genes was run in R (v4.5.x) through the DESeq2 package (v1.50.2) [21]. Normalization has been performed using a variance-stabilizing transformation (VST), which tends to stabilize the variance relative to the mean expression range and is more appropriate in RNA-seq count data before proceeding to downstream network and visualization analysis [21]. Lowly expressed genes were filtered to those with mean normalized counts < 10 in at least 2 samples, and 15,397 genes were kept for further analysis. The possibility of batch effects was alleviated by the nature of VST variance stabilization and confirmed by principal component analysis (PCA). All analyses were based on sample metadata such as disease status (RA vs HC) to align with the expression matrix.

### 2.2. Differential expression analysis

DESEQ2 (v1.50.2) was used in the R environment to perform the analysis of differential expression. A negative binomial generalized linear model was used to model the count data, with disease condition (RA vs HC) as the main design factor. Wald tests were used to estimate log2 fold changes and statistical significance, and then multiple testing was corrected using the Benjamini-Hochberg correction. An adjusted p-value < 0.05 and an absolute log2 fold change (|log2FC|) > 1 were used to define the differentially expressed genes. This threshold has been broadly accepted in transcriptomic studies and allows for prioritizing genes showing biologically significant changes in expression over those with only small differences. In the differential expression analysis, both upregulation and downregulation genes were identified, but further analyses were performed on the upregulated genes to identify activated ferroptosis- and cuproptosis-related pathways in RA. The results were analyzed with volcano, and MA plots were created with ggplot2 and EnhancedVolcano [21,22].

### 2.3. Weighted Gene Co-Expression Network Analysis (WGCNA)

Weighted gene co-expression network analysis (WGCNA) was performed with the WGCNA package (v1.74) of R [15] to identify gene modules related to rheumatoid arthritis (RA), in which the pickSoft Threshold function was used to identify the ideal soft-thresholding power (b = 9) to achieve scale-free topology (R2 > 0.85). Based on the expression data, variance-stabilizing transformation (VST)-normalized data were used to obtain a signed weighted adjacency matrix, which was then converted to a topological overlap matrix (TOM) for hierarchical clustering. Dynamic hybrid tree cutting with parameters minModuleSize = 30, deepSplit = 2, and mergeCutHeight = 0.25 identified gene modules. The eigengenes of each module (Mes), i.e., the first principal components of the module’s expression profiles, were then subjected to Pearson correlation analysis with the binary RA trait (RA = 1, HC = 0). The module with the largest absolute correlation coefficient with the RA phenotype was regarded as the key module. Lastly, candidate hub genes were selected based on predefined criteria, including high module membership (MM > 0.8) and high gene significance (GS > 0.5; p < 0.05). Genes not meeting these thresholds were excluded from subsequent analyses.

### 2.4. Identification of Ferroptosis- and Cuproptosis-related genes

The ferroptosis-related genes were downloaded from FerrDb V3 database containing ferroptosis-associated genes as divided into Driver genes, Suppressor genes, and Ferromarker genes. Some genes were found in more than one category, so a total of 1,519 non-redundant ferroptosis-related genes were ultimately obtained by selecting unique gene symbols. The full list of ferroptosis genes is shown in Supplementary Table S1 in S1 File (Excel file) [23,24]. The genes associated with cuproptosis were selected from published literature of genes involved in copper-induced cell death and cuproptosis regulation, with the important regulators of cuproptosis, FDX1, DLAT, and CDKN2A. To ensure maximal representation of the genes currently known to be involved in cuproptosis, additional genes were identified from studies published in the last three years (2022–2025). In total, 177 genes associated with cuproptosis were analyzed. The full list of genes, literature citations, and inclusion criteria are listed in Supplementary Table S2 in S2 File (Excel file) [25–27]. Venn diagram analysis showed that ferroptosis and cuproptosis shared common genes (~41 genes) [11,28]. Intersectively selected candidate genes were those that were significantly upregulated in the DEG list, in the top WGCNA module, and that overlapped the FRG/CRG gene lists. To determine the number of intersections, base R functions or the VennDiagram package were used [29].

### 2.5. Functional enrichment analysis

The analyses of functional enrichment were performed in R with the help of clusterProfiler (v4.16.0) [30]. The org. Hs. e.g., the db package was used to convert gene symbols to Entrez IDs [31]. The over-representation analysis (ORA) was used to perform gene ontology (GO) enrichment for biological processes, molecular functions, and cellular components, and the KEGG pathway analysis was performed at a significance level of p < 0.05 and adjusted p < 0.05 (Benjamini-Hochberg correction). Enrichplot and ggplot generated dot plots, bar plots, and network diagrams of the enrichment as described previously [30,32]. Reactome pathway enrichment was conducted using ReactomePA [33]. KEGG pathway enrichment was performed using clusterProfiler. A significance threshold of adjusted p < 0.05 was applied.

### 2.6. Immune infiltration analysis

A comprehensive set of immune cell gene signatures was selected based on established immunology literature and validated databases (IMMGEN, TCGA, ImmuneSig) [34]. The estimate of immune infiltration was performed using marker gene signatures for 18 distinct immune cells (Table 4) [35,36]. The infiltration score per immune cell type and sample was calculated as the average normalized expression of marker genes. The rank correlation coefficients for the expression of giants and immune cell infiltration were determined using Spearman’s correlation. T-tests compared the distributions of scores in RA and HC. Diversity indices, including Shannon (H = −∑ pi ln(pi)), were used to measure immune diversity.

### 2.7. Potential drug target identification

Potential therapeutic drug targets associated with the identified hub genes were examined using the Drug-Gene Interaction Database (DGIdb). This online database stores experimentally validated and predicted interactions of drugs and genes. These databases were queried using the hub genes identified by the integrative analysis to identify compounds with known or potential regulatory interactions. Data on drug name, regulatory approval, therapeutic indication, and interaction score were obtained for each gene-drug pair. The resulting interactions were filtered to prioritize approved drugs and compounds with higher interaction scores, enabling the identification of potential candidate therapeutic agents that can be repurposed to treat rheumatoid arthritis by interacting with the hub genes.

### 2.8. Single-cell RNA-seq analysis

The scRNA-seq data from rheumatoid arthritis synovial fluid were obtained in the GEO dataset GSE296117 and processed in the Seurat package in R to visualize the dimensionality reduction and cell distribution using DimPlot. The expression of hub genes (CDKN2A, SOD2, and FTH1) across cell groups was investigated using FeaturePlot, and DotPlot was used to show the mean expression and the proportion of cells expressing each gene in each cluster. VlnPlot was also applied to measure heterogeneity in gene expression across cell populations and to assess the distribution of heterogeneity of the hub gene expression at the single-cell level.

### 2.9. External validation

Candidate gene expression was validated in four independent GEO datasets: GSE55235 (GPL96, synovial tissue, n = 30), GSE55457 (GPL96, synovial tissue, n = 33), GSE77298 (GPL570, synovial tissue, n = 23), and GSE93272 (GPL570, synovial tissue, n = 275). Probe-to-gene mapping was done with platform-specific annotation packages (hgu133plus2.db for GPL570 and GPL annotation tables for GPL96). Furthermore, locally available count data for GSE169082 (PBMC, n = 7) were analyzed.

### 2.10. Visualization and statistical analysis

All statistical tests were carried out in a two-tailed test with a significance level of p < 0.05. Several comparisons have been corrected with the Benjamini-Hochberg approach. Ggplot2 (v3.5.2), pheatmap, EnhancedVolcano, and gridExtra were used to generate data visualization (volcano plots, heatmaps, boxplots of gene expression and ssGSEA scores, PCA plots, and dendrograms and matrices of module-trait correlations). All analyses have been performed in R (v4.5.2) with Bioconductor packages on a standard computing system.

## 3. Results

### 3.1. Identification of differentially expressed genes

**Fig 2:**
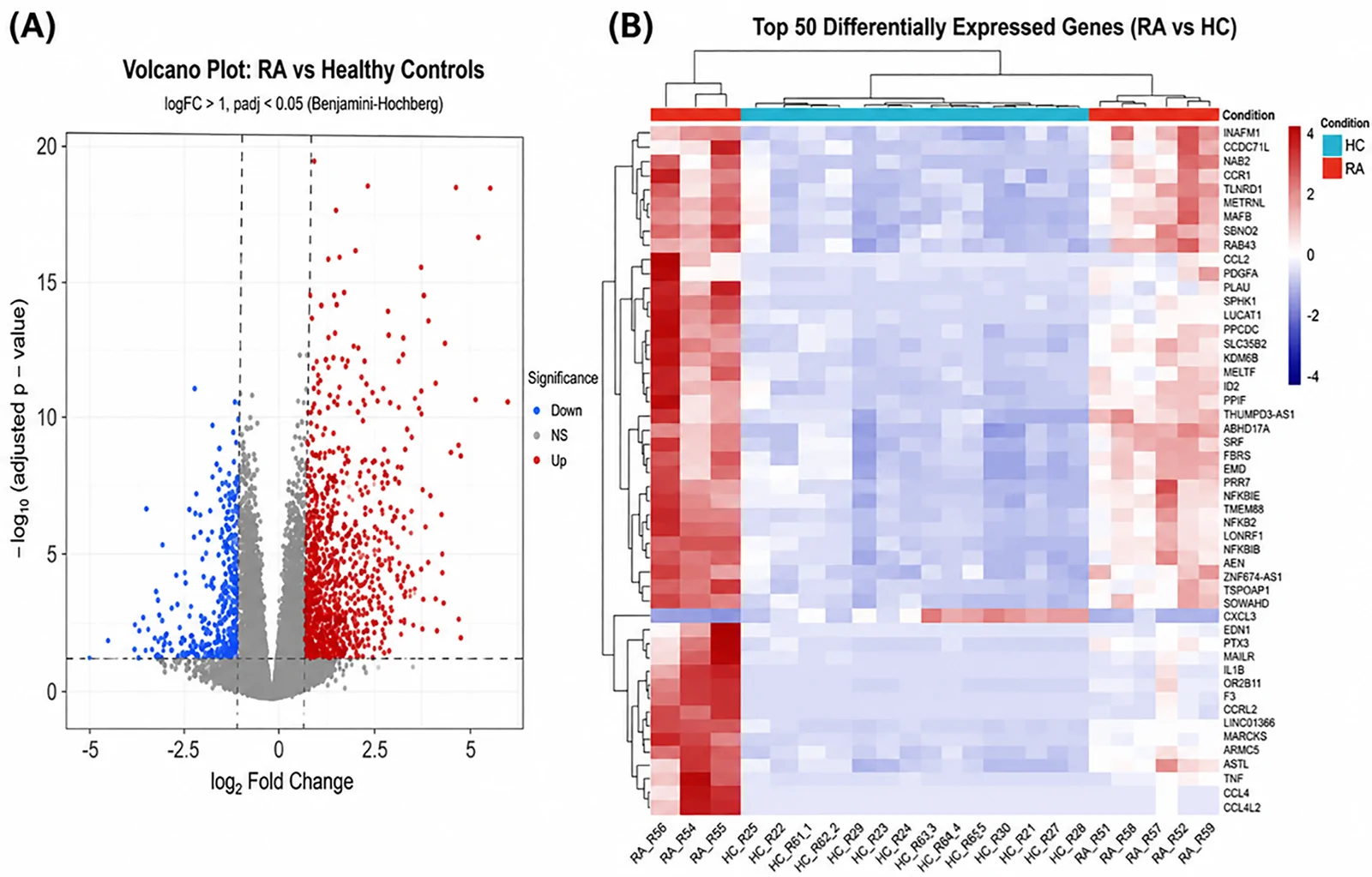
Differential gene expression analysis. (A) Volcano plot showing DEGs between rheumatoid arthritis (RA) and healthy control (HC) samples, with significantly up- and downregulated genes, respectively. (B) Heatmap of the top 50 DEGs across RA and HC samples, with color intensity representing relative expression levels.

The study’s workflow is shown in Fig 1. Differential expression analysis using DESeq2 identified 1,410 significantly differentially expressed genes (DEGs) between rheumatoid arthritis (RA) and healthy control (HC) CD14+ monocyte samples (adjusted p-value < 0.05, |log2 fold change| > 1). Among these, 1,200 genes were upregulated, and 210 were downregulated in RA (Fig 2). While the present study focused primarily on upregulated genes for downstream analyses, several downregulated genes may also have important biological roles in RA pathogenesis and warrant further investigation. The most strongly upregulated genes included key mediators of inflammation and cell death regulation, such as TNF (log2 FC = 6.61, adjusted p = 2.37 × 10^−159^), a pivotal pro-inflammatory cytokine; IL1B (log2 FC = 5.15, adjusted p = 2.76 × 10^−134^), a major regulator of inflammatory signaling; ARMC5 (log2 FC = 1.94, adjusted p = 5.58 × 10^−35^), associated with cellular stress responses; PPIF (log2 FC = 2.63, adjusted p = 2.69 × 10^−94^), a mitochondrial regulator implicated in ferroptosis through modulation of the mitochondrial permeability transition pore; and SPHK1 (log2 FC = 2.80, adjusted p = 3.04 × 10^−92^), which plays a role in sphingolipid metabolism and pro-inflammatory signaling.

### 3.2. Identification of Ferroptosis- and Cuproptosis-associated candidate genes

**Fig 3:**
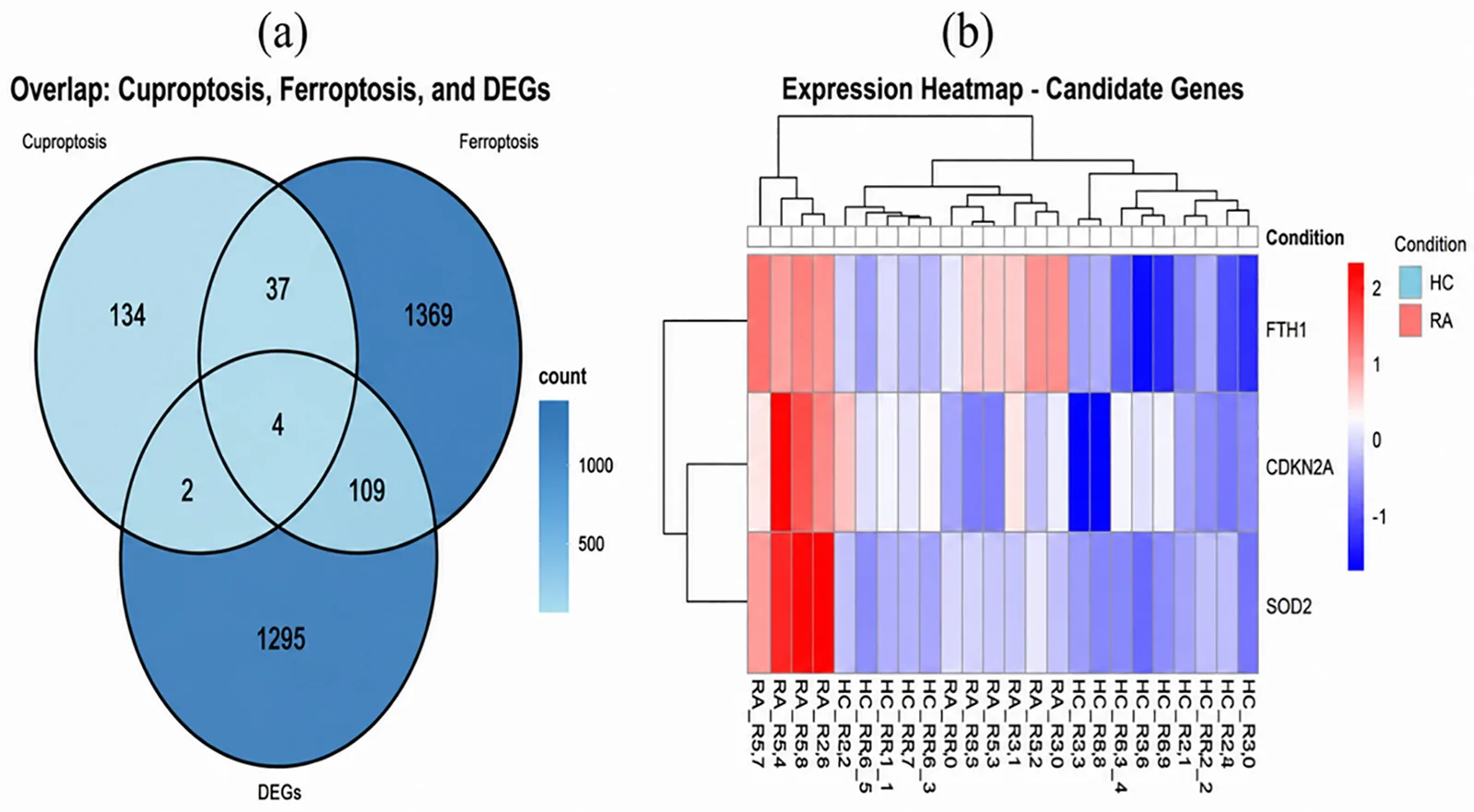
Candidate hub gene identification and expression. (a) Venn diagram showing overlap between DEGs, ferroptosis, and cuproptosis genes, highlighting candidate hub genes. (b) Heatmap of these hub genes across RA and HC samples; red indicates upregulation and blue indicates downregulation.

Among the selected ferroptosis-related genes (FRGs; n ≈ 1,519 on literature extensions), 113 were found to have a significant difference in their expression (113 adj. p-value < 0.05, 113 log2 FC > 1). Unipolar up-regulation prevailed with the functional providers of ferroptosis (Table 1). These activated genes indicate inflammatory priming and susceptibility to ferroptosis in RA monocytes; SLC7A11 is likely to be a reflection of compensatory antioxidant processes, and PPIF/PTGS2 is likely to play a role in lipid peroxidation pathways. Out of the selected cuproptosis-related genes (CRGs; n = 177), six were differentially expressed, with most of them being upregulated (Table 2). The intersection of upregulated DEGs with a list of ferroptosis-related genes and cuproptosis-related genes found four overlapping genes: *FTH1*, *SOD2*, *SCO2*, and *CDKN2A* (Fig 3). FTH1 (Ferritin Heavy Chain 1) is a dominant iron storage material that controls the intracellular iron concentrations and determines vulnerability to ferroptosis. The mitochondrial antioxidant enzyme SOD2 (Superoxide Dismutase 2) detoxifies the superoxide radical and regulates pathways involved in oxidative stress-induced ferroptosis and cuproptosis. The SCO2 (Synthesis of Cytochrome c Oxidase 2) is a copper-binding protein located in the mitochondria needed to assemble cytochrome c oxidase, which connects the copper metabolism to mitochondrial respiration and cuproptosis. CDKN2A (Cyclin-Dependent Kinase Inhibitor 2A; p16INK4a/p14ARF) is a tumor suppressor that regulates cell cycle arrest and cellular senescence, and it is also involved in metal dysregulation stress responses.

**Table 1:** Top Upregulated Ferroptosis-Related Genes in RA.

Gene	Function	log_2_FC	Adjusted p-value
*TNF*	Pro-inflammatory cytokine	6.614	2.37 × 10 ^−19^
*IL1B*	Inflammatory mediator	5.153	2.76 × 10 ^−16^
*PPIF*	Ferroptosis regulator (mitochondrial)	2.627	2.69 × 10 ^−13^
*SLC7A11*	Cystine transporter (xCT)	2.993	5.74 × 10 ^−5^
*NLRP3*	NLRP3 inflammasome component	2.363	1.14 × 10 ^−6^
*PTGS2*	Cyclooxygenase-2 (COX-2)	2.621	1.90 × 10 ^−6^

**Table 2:** Upregulated Cuproptosis-Related Genes in RA.

Gene	Function	log_2_FC	Adjusted p-value
*FTH1*	Ferritin heavy chain (iron storage)	1.019	5.83 × 10 ^−9^
*SOD2*	Superoxide dismutase 2 (mitochondrial antioxidant)	2.003	1.60 × 10 ^−7^
*SCO2*	Cytochrome c oxidase copper chaperone	1.329	4.02 × 10 ^−6^
*SLC40A1*	Iron exporter (ferroportin-1)	–1.996	1.95 × 10 ^−6^
*SLC25A33*	Mitochondrial carrier protein	1.111	8.68 × 10 ^−6^
*CDKN2A*	p16 tumor suppressor, senescence inducer	1.333	3.08 × 10 ^−2^

### 3.3. WGCNA Reveals Key Hub Genes Associated with RA

**Fig 4:**
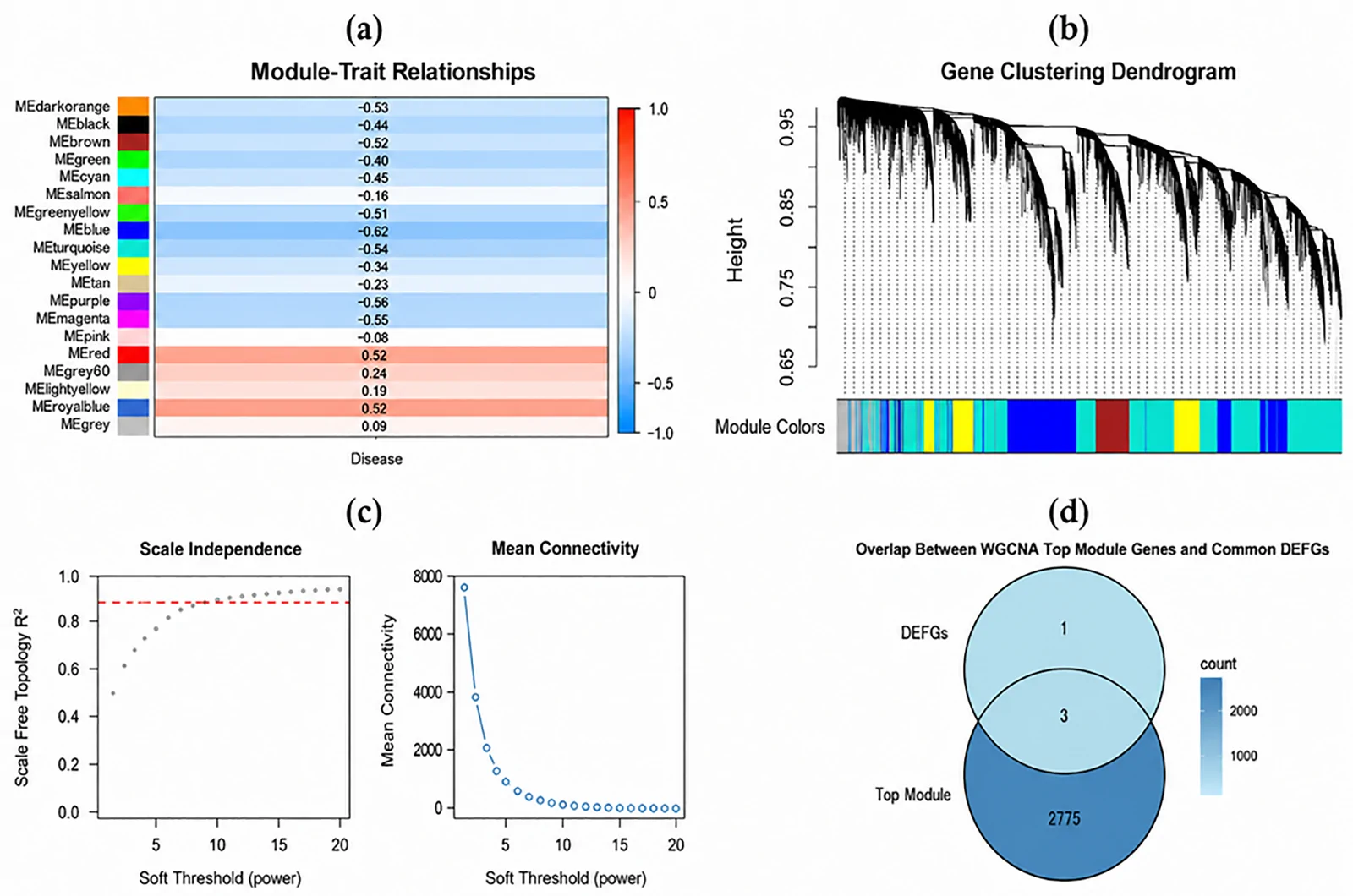
WGCNA analysis for identification of disease-associated modules and candidate genes. (a) Module–trait relationship heatmap showing correlations between gene co-expression modules and the disease phenotype. (b) Gene clustering dendrogram with module color assignments identified by WGCNA. (c) Determination of the soft-thresholding power based on the scale-free topology fit index and mean connectivity. (d) Venn diagram showing the overlap between genes from the top WGCNA module and common differentially expressed cuproptosis/ferroptosis-related genes, highlighting candidate genes for further analysis.

WGCNA identified 29 different co-expression modules based on the variance-stabilized gene expression matrix. The analysis of the module-trait correlation indicated that the blue module has the strongest, significantly positive relationship with the RA phenotype (Pearson correlation coefficient r = 0.65, p < 0.001; Fig 4.a. The module contained 2,778 genes, forming a big and well-connected transcriptional network. The blue module was filtered by selecting the 20 highest-hub genes, based on their module membership (MM) scores. Initially, four common genes (*FTH1*, *SOD2*, *SCO2*, and *CDKN2A*) were found after integrating the blue module genes with significantly upregulated DEGs and curated ferroptosis- and cuproptosis-related gene lists. The subsequent filtering with predefined module membership (MM) and gene significance (GS) criteria, however, identified only three genes, *FTH1*, *SOD2* and *CDKN2A*, as final hub genes, while *SCO2* did not meet the criteria and was excluded from further analyses. (Table 3) (Fig 4.d)). These three genes were found to have high intramodular connectivity (MM > 0.82), high trait relevance (GS > 0.68), and were highly upregulated in RA monocytes, placing them in the center of the RA-related transcriptional program.

**Table 3:** Candidate Hub Genes Identified from WGCNA Integration.

Gene	Module	Module Membership (MM)	Gene Significance (GS)	log_2_ fold change	Adjusted p-value
*FTH1*	Blue	0.85	0.72	1.329	4.02 × 10 ^−6^
*SOD2*	Blue	0.88	0.79	2.003	1.60 × 10 ^−7^
*CDKN2A*	Blue	0.82	0.68	1.333	3.08 × 10^−2^

MM: Module Membership (intramodular connectivity); GS: Gene Significance (correlation with RA trait).

### 3.4. Functional characteristics of candidate hub genes

**Fig 5:**
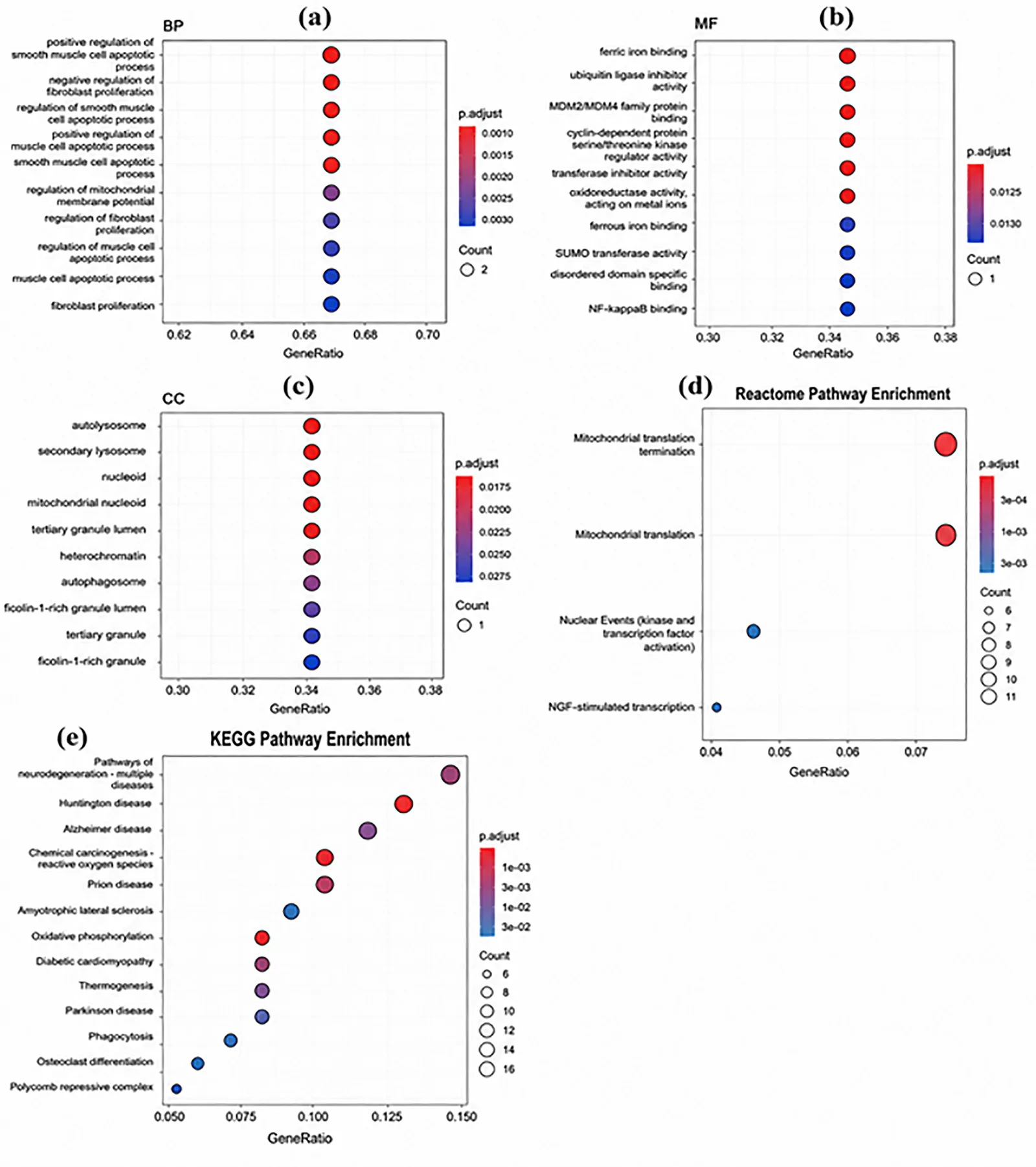
Functional enrichment analysis of DEGs showing significant (a) GO Biological Process (BP), (b) GO Molecular Function (MF), (c) GO Cellular Component (CC), (d) Reactome pathway, and (e) KEGG pathway enrichment results. Bubble size represents gene count, while color indicates the adjusted *P*-value (*p.adjust*).

The analysis of the hub genes *SOD2*, *CDKN2A*, and *FTH1* in terms of their functional enrichment using the Gene Ontology (GO) terms demonstrated that the three interact in a coordinated manner regarding the most important cellular processes related to intracellular organization, protein handling, mitochondrial maintenance, autophagy, redox regulation, iron-related functions, and immune functions. The genes were enriched (adjusted p < 0.05; gene ratio ≈ 0.33) in organelles and subcellular structures that are important in protein and vesicle traffic, such as autolysosome, secondary lysosome, nucleoid, mitochondrial nucleoid, and tertiary granule lumen, and in heterochromatin, autophagosome, and ficolin-1-rich granules, in the Cellular Component (CC) category (Fig 5). Under Molecular Function (MF) category, the hub genes were significantly enriched in ferric and ferrous iron binding, serine/threonine kinase inhibitor activity, SUMO transferase activity, ubiquitin ligase inhibitor activity, and oxidoreductase activity, which underscores their key roles in iron storage as well as iron homeostasis (through the contribution of FTH1), mitigation of oxidative stress (through the mitochondrial superoxide dismutase activity of SOD2), regulation of post-translational modifications, and cell cycle as these enrichment findings indicate that SOD2, CDKN2A, and FTH1 are important regulators of mitochondrial activity, protein quality control, vesicle transport, chromatin stability, autophagy, redox homeostasis, iron metabolism, and immune granule dynamics, which explains their integrated role in cellular homeostasis in response to oxidative and inflammatory stress that are important in disease pathogenesis. Reactome pathway analysis (Fig 5d) identified significant enrichment in mitochondrial translation, the citric acid (TCA) cycle, and respiratory electron transport, corroborating the role of mitochondrial dysfunction in RA monocytes. KEGG enrichment (Fig 5e) highlighted pathways in neurodegeneration, oxidative phosphorylation, and autophagy.

### 3.5. External validation, across cohorts

**Fig 6:**
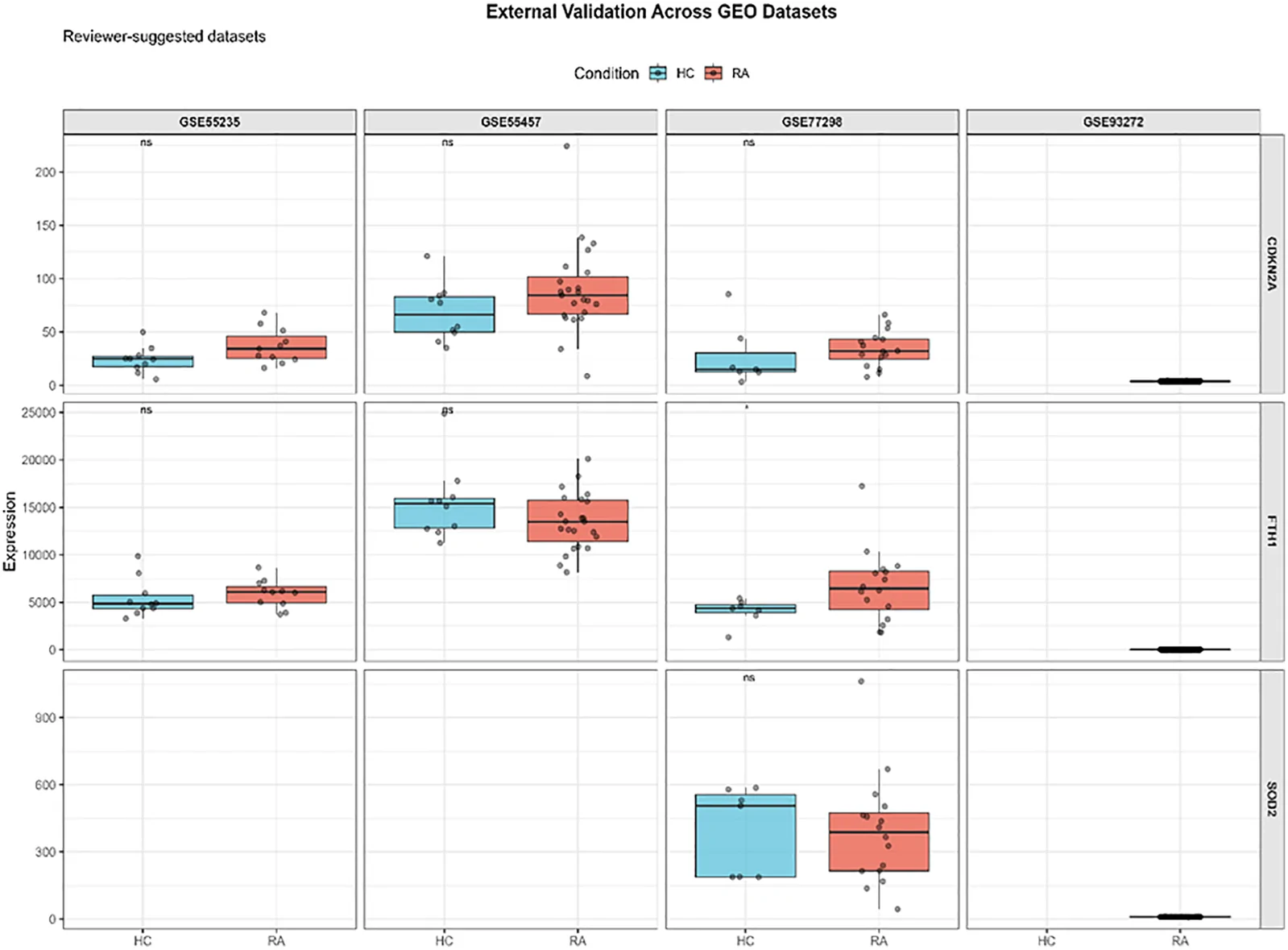
Validation of hub genes for candidates in independent GEO datasets. Expression levels of *CDKN2A*, *FTH1*, and *SOD2* were evaluated in GSE55235, GSE55457, GSE77298, GSE93272, and GSE169082. The results showed that the identified candidate genes were consistently expressed in independent RA cohorts, implying strong consistency of the candidate genes. HC: healthy controls; RA: rheumatoid arthritis.

Validation in four independent GEO datasets confirmed the upregulation of FTH1 and CDKN2A in RA synovial tissue across GSE55235 (HC = 10, RA = 11), GSE55457 (HC = 10, RA = 23), and GSE77298 (HC = 7, RA = 16) (Fig 6). SOD2 was represented on GSE77298 (GSE platform: GPL570) and validated, but was not represented on the GPL96 platform for GSE55235 and GSE55457. The upregulation of all three genes was further confirmed by analysis of GSE169082 (PBMC, HC = 3, RA = 4). The replication of these results in several independent cohorts and tissue sources (PBMC and synovium) enhances the overall replicability of our results.

### 3.6. Association of hub genes with immune cell infiltration

**Fig 7:**
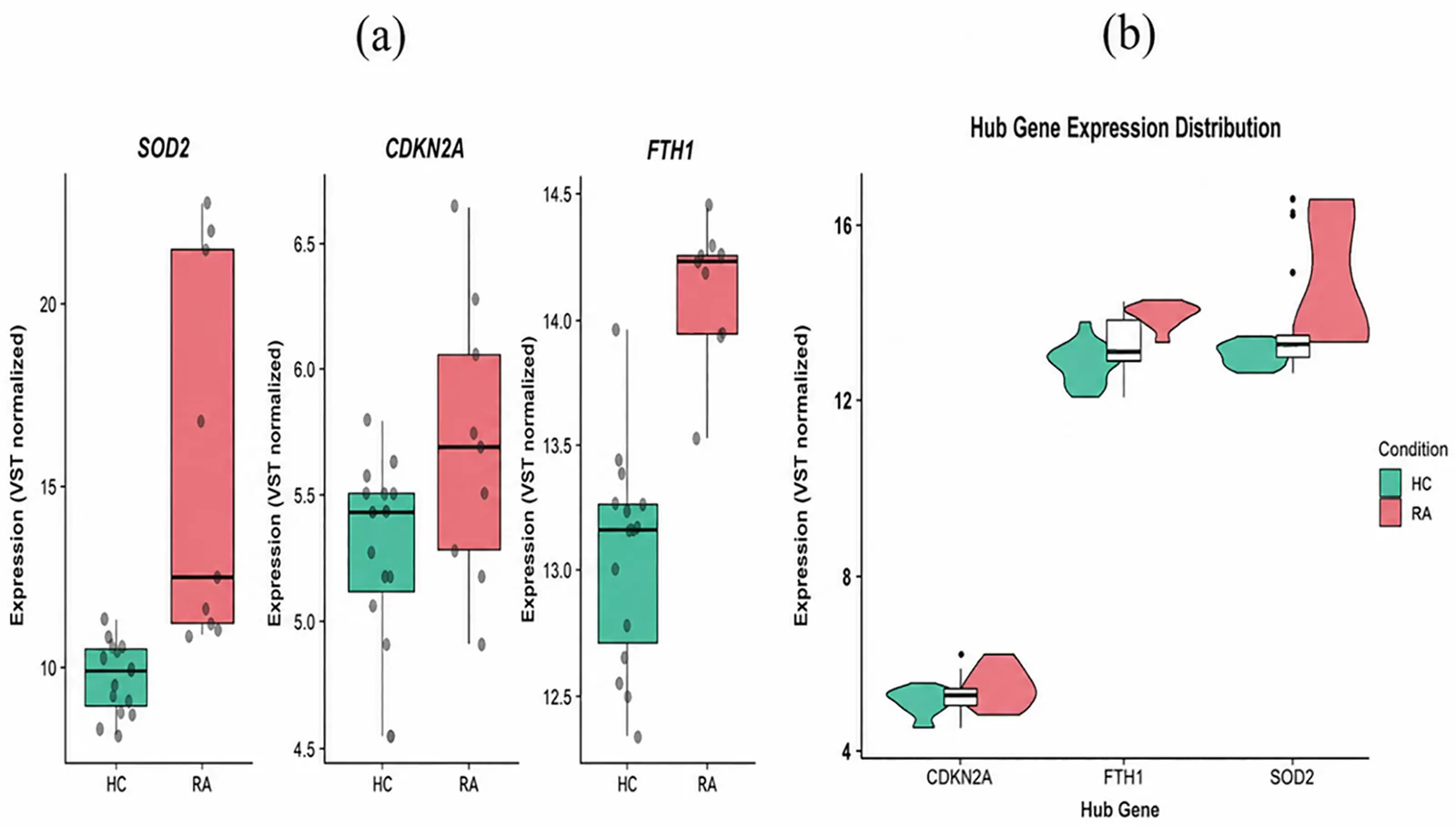
Expression distribution of identified hub genes in rheumatoid arthritis and healthy controls. (a) Boxplots showing normalized expression levels of the hub genes SOD2, CDKN2A, and FTH1 in rheumatoid arthritis (RA) and healthy control (HC) samples. (b) Violin plots with embedded boxplots illustrating the full expression density distributions of the hub genes across conditions, highlighting differential expression patterns between RA and HC samples.

It selected a total of 18 population immunology-based immune cell signatures based on the current literature and validated immunology databases Table 4. The differences in the levels of the hub genes (*SOD2*, *CDKN2A*, and *FTH1*) between rheumatoid arthritis (RA) and healthy controls (HC) were identified using a two-sample t-test. The results showed that the FTH1 and SOD2 were significantly elevated in RA compared to HC. FTH1 was the most correlated to RA, and the average of the expression was 14.11 in RA and 13.05 in HC (t = 7.39, p = 2.27 x 10^−7^). Similarly, the expression of SOD2 in RA (mean = 14.74) was significantly higher than that of HC (mean = 13.26) (t = 3.33, p = 0.0098). Instead, it showed that there was minimal variation of RA in CDKN2A (mean = 5.46) relative to HC (mean = 5.11), which was not significantly different (p = 0.0627) (Tables 4 and 5) (Fig 7a–b). These findings imply that FTH1 and SOD2 may play important roles in RA pathogenesis.

**Table 4:** Eighteen immune cell types analyzed with their respective marker genes detected in the dataset.

Immune Cell Type	Markers Found	Total Markers	Key Genes
CD8 ⁺ T cells	5	5	*GZMA, GZMB, CD8B, PRF1*
CD4 ⁺ T cells	4	4	*IL7R, IL2RA, CD4*
Regulatory T cells	4	4	*CTLA4, IL2RA, FOXP3*
B cells	4	4	*CD79B, CD79A, MS4A1*
Plasma cells	4	4	*IGHG1, CD27, MZB1*
Memory B cells	4	4	*IGHG1, CD27, IGHG2*
Macrophages	4	4	*IL1B, CD68, CSF1R*
M1 Macrophages	3	4	*IL1B, IL6, TNF*
M2 Macrophages	4	4	*IL10, MRC1, CD163*
Monocytes	4	4	*S100A8, LYZ, S100A9*
Dendritic cells	3	4	*HLA-DRA, CD80, CD86*
Neutrophils	4	4	*S100A8, LYZ, FCGR3B*
NK cells	4	4	*GZMB, GNLY, NCAM1*
Eosinophils	3	3	*PRG2, RNASE2, EPX*
Mast cells	2	3	*MS4A2, KIT*
Th1 cells	2	4	*TBX21, TNF*
Th2 cells	1	4	*GATA3*
Th17 cells	0	4	*Synthetic scores*

**Table 5:** Statistical comparison of hub genes expression between RA and healthy control samples. Significance indicated at p < 0.05 (*).

Gene	Mean (RA)	Mean (HC)	SD (RA)	SD (HC)	t-statistic	p-value
*SOD2*	14.74	13.26	1.32	0.24	3.33	0.0098*
*CDKN2A*	5.46	5.11	0.45	0.30	2.04	0.0627
*FTH1*	14.11	13.05	0.27	0.43	7.39	2.27 × 10 ^−7^*

**Fig 8:**
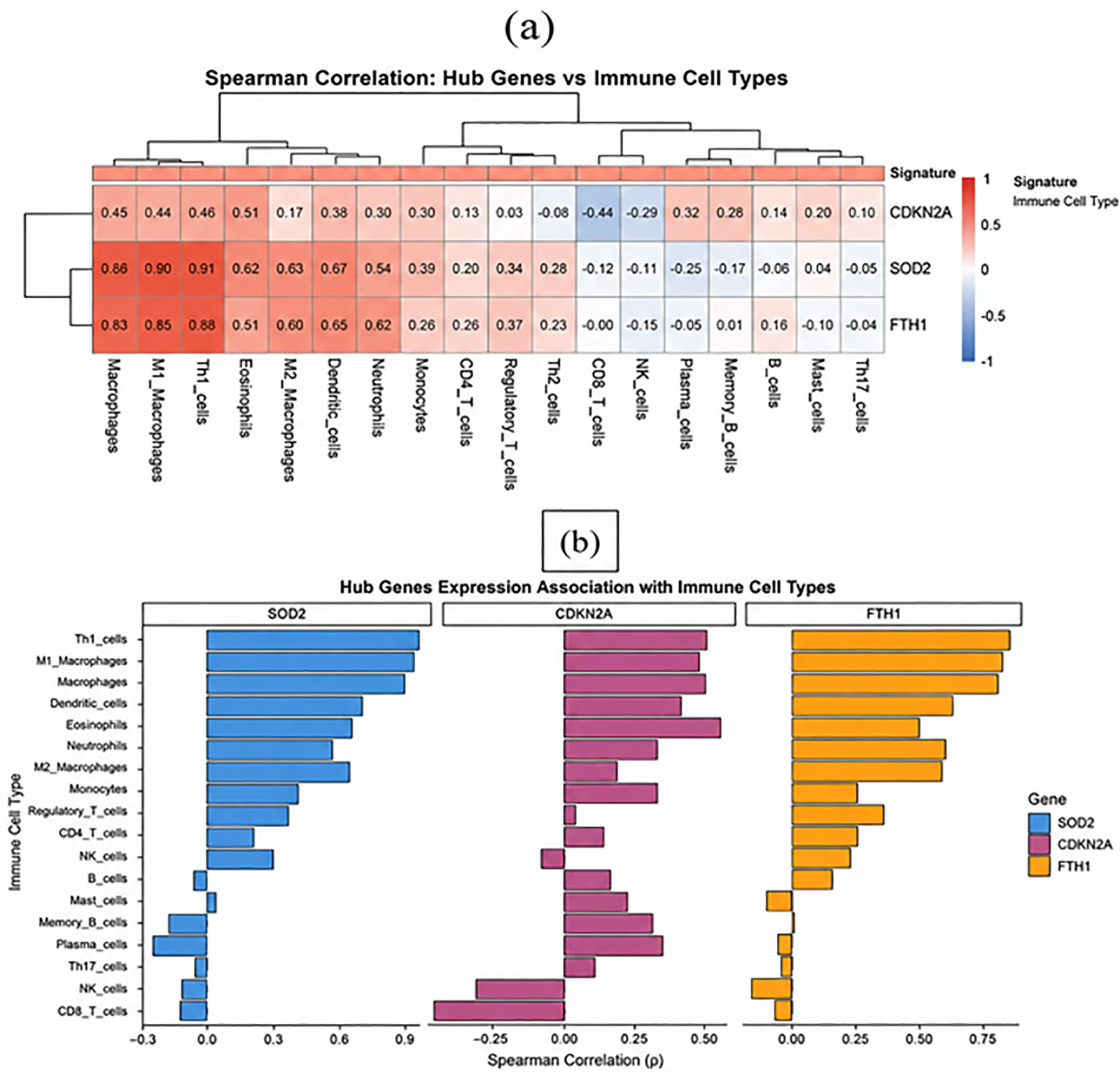
Correlation between hub genes and immune cell infiltration signatures. (a) Spearman correlation heatmap showing the associations between hub genes and immune cell type signatures. Color intensity represents the correlation coefficient ranging from −1 (blue) to +1 (red), with each cell indicating the strength of the correlation. (b) Ranked bar plots illustrating the correlations between each hub gene (*SOD2*, *CDKN2A*, and *FTH1*) and immune cell types, highlighting distinct immune association patterns, with FTH1 showing the strongest correlations with myeloid cell populations.

The Spearman correlation analysis revealed individual associations between the hub gene expression and immune cell populations. SOD2 was positively correlated with CD8+ T cells (r ≈ 0.3–0.5), suggesting that it is involved in the activity of cytotoxic lymphocytes. CDKN2A was linked to Th1 and Th2 cell differentiation markers, consistent with its regulation of the cell cycle in helper T cells. Conversely, FTH1 showed the highest correlations with myeloid cell subsets, such as macrophages, monocytes, and dendritic cells, suggesting a potential interconnection between iron metabolism and innate immunity (Fig 8a–b).

**Fig 9:**
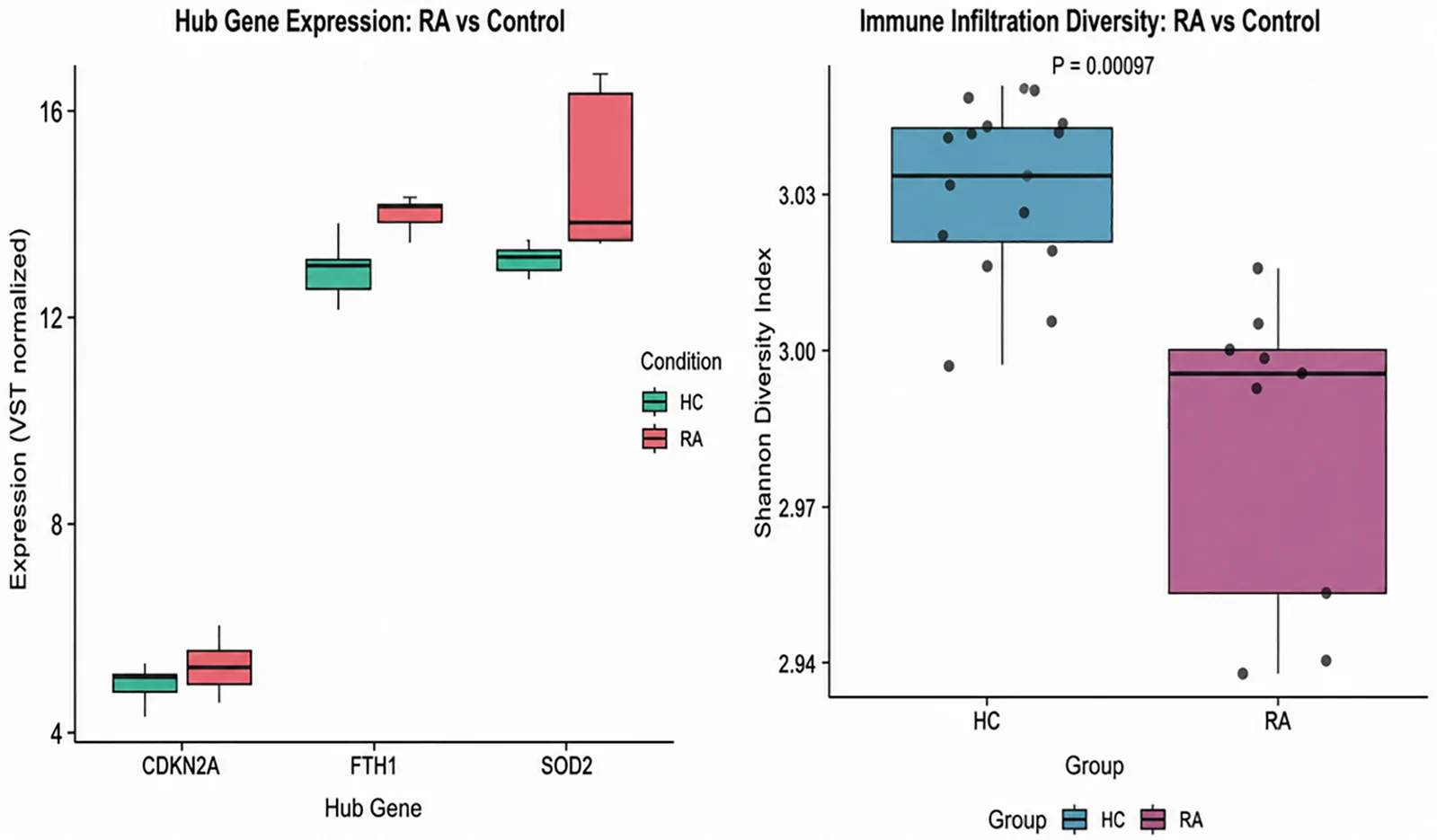
Hub gene expression and immune infiltration diversity in rheumatoid arthritis. (a) Box plots showing normalized expression levels of the hub genes *CDKN2A*, *FTH1*, and *SOD2* in rheumatoid arthritis (RA) and healthy control (HC) samples. (b): Box plot of the Shannon diversity index indicating significantly reduced immune infiltration diversity in RA compared to HC (P = 0.00097).

Combinations of hub gene expression data and immune infiltration analysis revealed distinct immune patterns related to disease status. The presence of elevated *FTH1* in RA was associated with greater infiltration of myeloid lineage cells and with *SOD2* expression and cytotoxic lymphocyte markers (Fig 9(a)). On the whole, these findings indicate that abnormalities in metal-dependent cell death signaling may contribute to the expansion of certain populations of immune cells in rheumatoid arthritis. To further assess the complexity of the immune ecosystem, the Shannon diversity index was computed for each sample. The index was slightly lower in RA than in HC (p = 0.053), suggesting a tendency toward lower immune diversity as evidenced by the Th17/Treg imbalance and macrophage polarization that characterizes RA (Fig 9(b)).

### 3.7. Single-cell validation of hub gene expression

**Fig 10:**
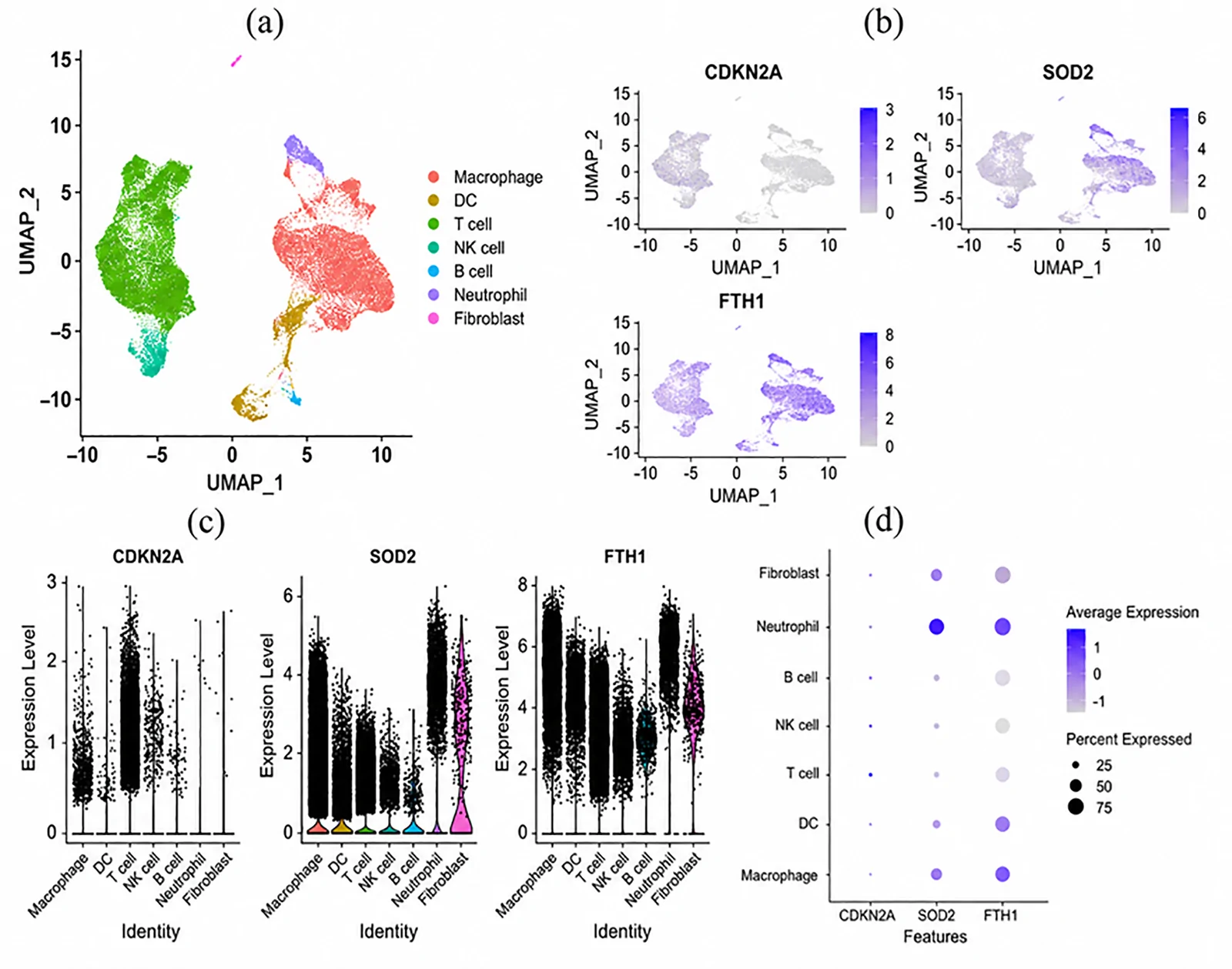
Single-cell RNA-seq analysis of hub gene expression in rheumatoid arthritis synovial fluid (GSE296117). (a) UMAP visualization showing the distribution of major cell populations, including macrophages, dendritic cells (DCs), T cells, NK cells, B cells, neutrophils, and fibroblasts.(b) Feature plots illustrating the expression patterns of the hub genes *CDKN2A*, *SOD2*, and *FTH1* across the identified cell clusters. (c) Violin plots displaying the distribution and variability of gene expression levels for the hub genes across different cell types. (d) Dot plot summarizing the average expression levels and the proportion of cells expressing each hub gene within the major immune and stromal cell populations.

Single-cell expression profiling of the identified hub genes in rheumatoid arthritis (RA) synovial fluid (GSE296117 dataset) has revealed differences in distribution patterns among the major cell populations, including macrophages, dendritic cells (DCs), T cells, NK cells, B cells, neutrophils, and fibroblasts (Fig 10a–b). The expression of *CDKN2A* was highest in macrophages (medians of approximately ~2–2.5 with wide distributions up to approximately 3) and significant in T cells and fibroblasts (medians of approximately ~1.5–2), but minimal in DCs, NK cells, B cells, and neutrophils (medians of approximately 0–1). *SOD2* showed high macrophage expression (median-5) and fibroblasts (especially median −5 with a high-expressing subpopulation of 5–6), with moderate expression in T cells, DCs, NK cells, and B cells (median-4). Ubiquitous cell-type expression (median typically ~4–7) was seen in *FTH1*, highest in macrophages (median 6–7, high density, to ~8+) and fibroblasts (median 5–6, significant subpopulation at ~6–7), then moderately-highly in T cells, DCs, NK cells and B cells, and relatively lower (but still substantial) in neutrophils (median 3–4) (Fig 10.c-d). These patterns indicate that overall hub genes are expressed in the myeloid (macrophage-enriched) and stromal (fibroblast) compartments of the RA synovial microenvironment, contributing to their functions in oxidative stress management, iron homeostasis, and regulated cell death pathways in disease-relevant cells.

### 3.8. Identification of candidate therapeutic compounds

The drug-target interaction analysis identified several approved drugs predicted to interact with the hub genes *CDKN2A*, *SOD2*, and *FTH1*. Most interactions were identified with *CDKN2A*, which is also targeted by anticancer therapies such as ribociclib, vemurafenib, cobimetinib, dabrafenib, trametinib, cetuximab, panitumumab, gemcitabine, paclitaxel, and letrozole, indicating the pharmacological relevance of CDKN2A-regulated networks, including cell-cycle regulation and MAPK signaling. The best score in the interaction was shown by ribociclib (0.52). In addition, the immunosuppressive mTOR inhibitors everolimus and sirolimus have been reported to be linked to CDKN2A, which may have immunomodulatory consequences. In the case of SOD2, several authorized drugs were found, among which is methotrexate, a cornerstone DMARD in the management of RA. Other compounds included valproic acid, paclitaxel, docetaxel, doxorubicin, daunorubicin, and asparaginase, with asparaginase having the highest interaction score (0.73). On the other hand, FTH1 primarily interacted with Ferritarg P, a ferritin-targeting agent yet to be clinically approved, which had an extremely high interaction score (52.2) (Table 6). Together, these results indicate that the identified hub genes could be potential pharmacologically relevant targets, and they offer possible drug repurposing in rheumatoid arthritis.

**Table 6:** Potential Therapeutic Drugs.

Gene	Drug	Regulatory approval	Indication	Interaction score
*CDKN2A*	RIBOCICLIB	Approved	antineoplastic agent	0.517895
*CDKN2A*	PACLITAXEL	Approved	for treatment of peripheral arterial disease (PAD), DMARD, anti-inflammatory agent, antineoplastic agent	0.033899
*CDKN2A*	VEMURAFENIB	Approved	antineoplastic agent	0.201559
*CDKN2A*	CETUXIMAB	Approved	antineoplastic agent	0.10078
*CDKN2A*	SAPANISERTIB	Approved	antineoplastic agent	0.162124
*CDKN2A*	COBIMETINIB	Approved	antineoplastic agent	0.196255
*CDKN2A*	GEMCITABINE	Approved	antineoplastic agent	0.040531
*CDKN2A*	PANITUMUMAB	Approved	antineoplastic agent	0.093221
*CDKN2A*	EVEROLIMUS	Approved	Immunosuppressant	0.109672
*CDKN2A*	LETROZOLE	Approved	antineoplastic agent	0.103579
*CDKN2A*	TRAMETINIB DIMETHYL SULFOXIDE	Approved	antineoplastic agent	0.27768
*CDKN2A*	DABRAFENIB	Approved	antineoplastic agent	0.276211
*CDKN2A*	SIROLIMUS	Approved	for the treatment of wet age-related macular degeneration, an immunosuppressant	0.06429
*SOD2*	METHOTREXATE	Approved	DMARD	0.079097
*SOD2*	ASPARAGINASE	Approved		0.725053
*SOD2*	DOCETAXEL ANHYDROUS	Approved	antineoplastic agent	0.104827
*SOD2*	PACLITAXEL	Approved	for treatment of peripheral arterial disease (PAD), DMARD, anti-inflammatory agent, antineoplastic agent	0.079097
*SOD2*	VALPROIC ACID	Approved	Anticonvulsants, for the treatment of basal cell carcinoma, are anticonvulsants	0.152643
*SOD2*	DOXORUBICIN HYDROCHLORIDE	Approved	antineoplastic agent	0.058788
*SOD2*	DAUNORUBICIN LIPOSOMAL	Approved	antineoplastic agent	0.095611
*FTH1*	FERRITARG P	Not Approved		52.2

## 4. Discussion

The current integrative analysis is based on differential expression, weighted gene co-expression network analysis (WGCNA), functional annotation, and immune infiltration deconvolution to comprehend metal-dependent cell death in peripheral CD14+ monocytes of rheumatoid arthritis (RA) patients. The DESeq2 package [21] was used to identify significantly changed transcripts (1410), most of which were upregulated, including inflammatory and oxidative stress mediators (*TNF, IL1B,* and *SPHK1*). Such findings are consistent with the already known pro-inflammatory microenvironment in RA monocytes [20,37] and are a basis for further network modeling. The modules identified by weighted gene co-expression analysis using the WGCNA package [15] comprised 29, with the blue module showing the highest positive correlation with the RA trait (r = 0.65, p < 0.001). Additional support for the relevance of this module to metal-dependent regulated cell death (RCD) included its enrichment in oxidative metabolism, mitochondrial dysfunction, and cell death processes.

The integration of module membership and ferroptosis and cuproptosis curated gene sets identified three hub genes (*FTH1, SOD2*, and *CDKN2A*) with significant increases in RA. Although *SCO2* was initially identified in the overlap analysis, it did not exhibit sufficient network connectivity within the RA-associated module and was therefore excluded based on the predefined WGCNA hub gene selection criteria. These genes are centrally located in the network and exhibit high correlations between modified immune infiltration patterns. Their function is to connect the processes of iron and copper metabolism with the redox system and the cellular cycle, in line with recent models suggesting that dysregulated metal management may be a cause of RA pathogenesis [8,38]. The significant enrichment across all Gene Ontology domains was revealed by functional enrichment analysis conducted using clusterProfiler and enrichplot packages [30]. Compounds that were enriched in cells included autolysosomes, mitochondrial nucleoids, heterochromatin, and immune granule compartments, and this showed functions in mitochondrial functions, autophagy, and chromatin dynamics. Terms of molecular function were identified as iron binding, oxidoreductase activity, and kinase inhibitor activity, which are associated with the biological activity of *FTH1*, *SOD2*, or *CDKN2A*. Taken together, this evidence suggests that disruptions in metal ion homeostasis, ROS detoxification, and proteostasis may also contribute to the monocyte dysfunction in RA.

The analysis was performed using single-sample gene set enrichment analysis (ssGSEA) based on 18 immune signatures derived from literature and data sources, including IMMGEN [34]. The scores were calculated as the mean normalized expression of marker genes, and statistical analysis was performed using ggplot2 [32]. The patterns of Spearman correlations between the expression of hub genes and immune cell scores showed some differences: *FTH1* was strongly correlated with myeloid populations (macrophages, monocytes, dendritic cells), suggesting that the dynamics of iron storage can affect innate immune activation [12]. *SOD2* was positively correlated with CD8+ T cells, consistent with mitochondrial oxidative stress regulation in cytotoxic lymphocytes [39]. *CDKN2A* was also associated with Th1/Th2 differentiation, which is consistent with its cell cycle and senescence activities [40]. Significant upregulation of FTH1 (p = 2.27 x 10^7^) and SOD2 (p = 0.0098), but a nonsignificant trend was confirmed in RA versus healthy controls (differential expression in hub genes as t-tests with Benjamini-Hochberg adjustment). The results implicate *FTH1* and *SOD2* as immune remodeling factors in RA, which may act through ferroptotic or cuproptotic pathways, regulating monocyte and lymphocyte phenotype. The calculated Shannon diversity indices of the immune scores indicated a slight decrease in immune ecosystem complexity in RA (p = 0.053), in line with previous studies reporting skewed Th17/Treg ratio and macrophage polarisation [41,42].

Collectively, WGCNA, DESeq2, and immune profiling integration constitute a network in which metal ion metabolism and oxidative stress are dysregulated to promote the formation of pro-inflammatory monocyte states and to coordinate with some adaptive immune subsets. The role of *FTH1*, *SOD2* and *CDKN2A* as core nodes in this network is mechanistic in nature: *FTH1* regulates intracellular iron buffering and might leave cells vulnerable to ferroptotic death in situations of iron overloading [8,10] *SOD2* counteracts mitochondrial ROS and might dampen or amplify ferroptotic/cuproptotic signaling depending on circumstance [43] and *CDKN2A* promotes senescence and may leave cells vulnerable to metallon-induced proteotoxic stress [44]. Combined, these genes are candidate biomarkers and treatment targets of interventions to restore metal homeostasis or alter RCD pathways in RA.

Combining WGCNA, DESeq2, and analysis of immune infiltration reveals a network in which impaired metal ion homeostasis and oxidative stress enhance inflammatory monocyte phenotypes and interact with adaptive immune cells. In this network, *FTH1*, *SOD2*, and *CDKN2A* are the most important nodes, providing a mechanistic understanding of the disease processes. *FTH1* controls iron storage within cells and can condition cells to ferroptosis in cases of iron buildup. *SOD2* regulates mitochondrial ROS levels and can either restrain or enhance ferroptotic and cuproptotic signaling depending on the cellular context. Meanwhile, cellular senescence is regulated by *CDKN2A* and may contribute to the susceptibility to metal-based proteotoxic stress. These genes will collectively serve as useful biomarkers and possible candidate therapeutic targets to implement strategies to restore metal homeostasis or regulate regulated cell death pathways in rheumatoid arthritis.

Our results are consistent with and build upon current literature. Ferroptosis has also been implicated in modulating the behavior of fibroblast-like synovial cells (FLS) and osteoarthritis destruction. Changes in the distribution of iron in synovial tissue have been reported [8,45], whereas cuproptosis processes are less investigated in RA but are biologically relevant due to observed copper imbalances and mitochondrial dysfunction in immune cells of patients [38,46]. FTH1-myeloid infiltration correlation supports the hypothesis that iron-handling processes regulate monocyte-macrophage differentiation and the secretion of inflammatory cytokines [12,47]. In addition, the correlation between SOD2 and CD8+ T cells suggests a link between oxidative stress defense and cytotoxic immune responses that could be involved in tissue damage or regulatory T cell exhaustion [39]. The association of *CDKN2A* with helper T cell subsets makes cell-cycle checkpoints and senescence a possible cause of unbalanced adaptive immunity in RA [40,44]

Several limitations warrant discussion. The analysis is based on peripheral blood monocytes rather than synovial tissue, which may not fully capture the joint microenvironment [20,37]. Sample size was modest (n = 24), potentially limiting power to detect subtle effects. Immune infiltration scores derived from bulk RNA co-expressed genes may be confounded by and require validation with orthogonal methods such as flow cytometry or single-cell RNA-seq [34,47]. Finally, while the curated ferroptosis and cuproptosis gene lists are comprehensive, novel regulators may be missing, and direct functional assays will be needed to confirm the roles of identified hub genes in metal-dependent RCD. The present study primarily focused on upregulated genes to identify activated ferroptosis- and cuproptosis-related pathways in RA; downregulated genes may also contribute to disease pathogenesis through the loss of protective or inhibitory regulatory mechanisms. Further investigations that involve the upregulation and downregulation of genes might give a wider picture of the pathways of metal-mediated cell death in RA. Our integrative analysis also identified genes common to both ferroptosis- and cuproptosis-related pathways, but the current study is focused on transcriptomic and network-level associations, and is not directly mechanistic for the functional interactions between these cell death mechanisms. Thus, more experimental studies are needed to confirm these findings and to elucidate the biological correlation between ferroptosis and cuproptosis in RA patients.

Single-cell profiling (GSE296117) indicated that *FTH1*, *SOD2*, and $*CDKN2A* were highly expressed by RA synovial macrophages and fibroblasts (median 4–7, 8+ in macrophages and fibroblasts, respectively). To ensure the robustness of our findings, we validated the hub genes identified in our study in multiple independent GEO cohorts and found the expression patterns to be similar across synovial tissue and PBMC, indicating the reproducibility and generalizability of our identified candidate genes. The pattern is biologically important in a mechanical sense: macrophages and fibroblast-like synoviocytes (FLS) are the main drivers of RA pathophysiology through cytokine production, matrix degradation, and osteoclast stimulation [4]. These metal-dependent cell death regulators are highly expressed in these cells, indicating that ferroptosis and cuproptosis can serve as tissue-resident checkpoints that regulate the activation of pathogenic cells, and that their dysregulation can lead to unchecked synovial inflammation.

The analysis of drug-gene interactions revealed some clinically significant compounds for which immediate therapeutic avenues are available. The clinical gold standard DMARD in RA, methotrexate [48], was found to interact with *SOD2*, suggesting a mechanistic overlap between its traditional immunosuppression activity and the regulation of oxidative cell death pathways. The identification of mTOR inhibitors (everolimus, sirolimus) that inhibit *CDKN2A* signaling suggests that immune checkpoint control can cooperate with cell cycle/senescence regulation.

Most interestingly, Ferritarg P, a ferritin-targeting compound with an impractically high interaction score (52.2) with *FTH1*, is an unapproved biologically promising agent for ferroptosis regulation in RA, and warrants further research. The mechanistic similarities between RA monocyte hyperactivation and oncogenic signaling pathways are highlighted by approved anticancer agents (ribociclib, vemurafenib, cobimetinib, dabrafenib, trametinib) that interact with *CDKN2A*, which are increasingly being viewed in immunology [49]. These are some of the compounds that can be considered as repurposing candidates in RA by targeting cellular stress response pathways.

Future experiments should examine the causal contributions of *FTH1*, *SOD2*, and *CDKN2A* to monocyte ferroptosis/cuproptosis, including CRISPR-based perturbations, lipid peroxidation, mitochondrial integrity, and cytokine responses. Their role in clinical use as biomarkers can be identified by examining their relationships with measures of disease severity and treatment responses. In addition, the effects of metal chelators or RCD modulators on RA models can be investigated to develop therapeutic approaches to reduce synovial inflammation and joint destruction.

## 5. Conclusion

This study used an integrative transcriptomic method to analyze peripheral monocytes from patients with rheumatoid arthritis and found a disease-related co-expression module enriched with metal-dependent regulated cell death pathways. The overlapping of this module and curated ferroptosis and cuproptosis gene sets identified three major hub genes, namely, *FTH1*, *SOD2*, and CDKN2A, that are highly upregulated in RA and that play central positions in the gene network. Their roles in iron metabolism, oxidative stress responses, autophagy, and cell-cycle control were confirmed by functional enrichment analysis, indicating that maladapted metal homeostasis and oxidative damage contribute to the pathophysiology of RA. Immune cell infiltration analysis also suggested that these hub genes were closely associated with both the lymphoid and myeloid immune cell subsets. These findings identify novel mechanistic concepts underlying the association among ferroptosis, cuproptosis, and immune dysregulation in RA and suggest targeted genes as possible biomarkers and therapeutic options. The external validation in independent cohorts further strengthens the potential relevance and robustness of these candidate genes in the pathogenesis of RA. Their clinical relevance will require future research using large patient cohorts and experimental systems.

## Supporting Information

S1 File Full list of ferroptosis-associated genes. (XLSX)

S2 File Full list of cuproptosis-associated genes. (CSV)
